# Supplementary material for: LIVE-PAINT allows super-resolution microscopy inside living cells using reversible peptide-protein interactions
Source: Commun Biol. 2020 Aug 20;3:458. doi: 10.1038/s42003-020-01188-6 (PMC7441314; doi:10.1038/s42003-020-01188-6)
Supplement: Supplementary file 1 — Supplementary Information [file 42003_2020_1188_MOESM1_ESM.pdf]

Supplementary Figures

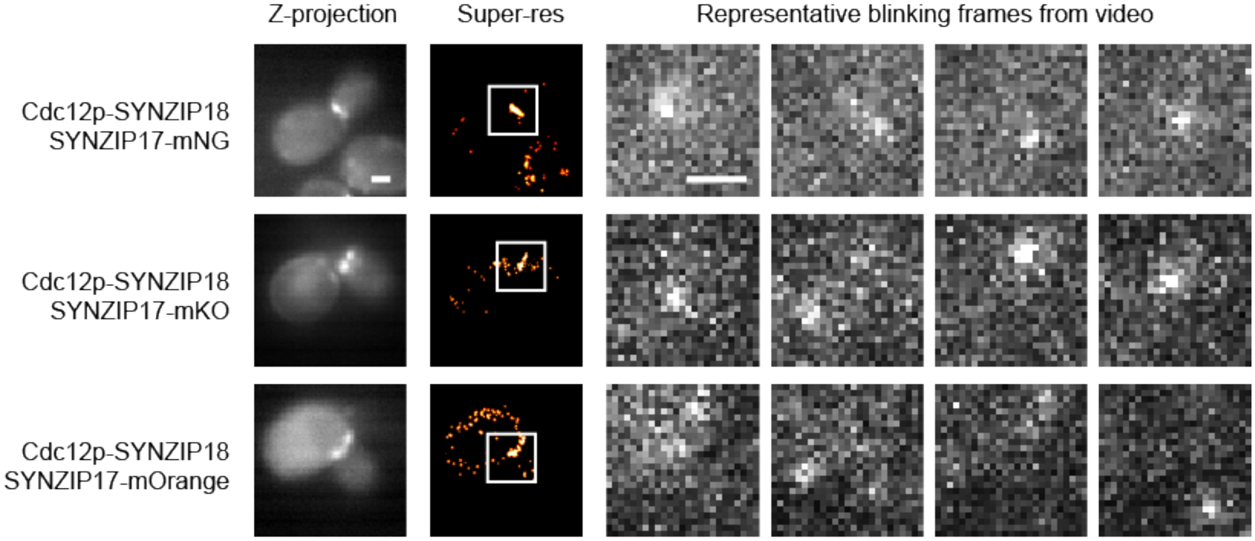

Supplementary Figure 1. LIVE-PAINT can be performed with different fluorescent proteins. LIVE-PAINT was performed using the SYNZIP17-18 interaction pair to tag Cdc12p and three different fluorescent proteins: mNG, mKO, and mOrange, as indicated. (Left) Z-projections showing the average fluorescence signal for each video, calculated by integrating the average intensity over the entire video. (Middle) Super-resolution images for each video. The white box corresponds to the cropped region shown in the "representative blinking frames from video" section at right. (Right) Representative frames from the video, showing bright "blinks" in different locations. All images were constructed from videos collected for 1,000 frames, with a 50 ms exposure per frame. Number of localization events: mNG: 531; mKO: 280; mOrange: 154. Scale bars are 1 μm. The mNG images were obtained with a 488 nm laser, using a power of 3 W/cm^2, while the mKO and mOrange images were obtained with a 561 nm laser, using a power density of 50 W/cm^2 (see methods).

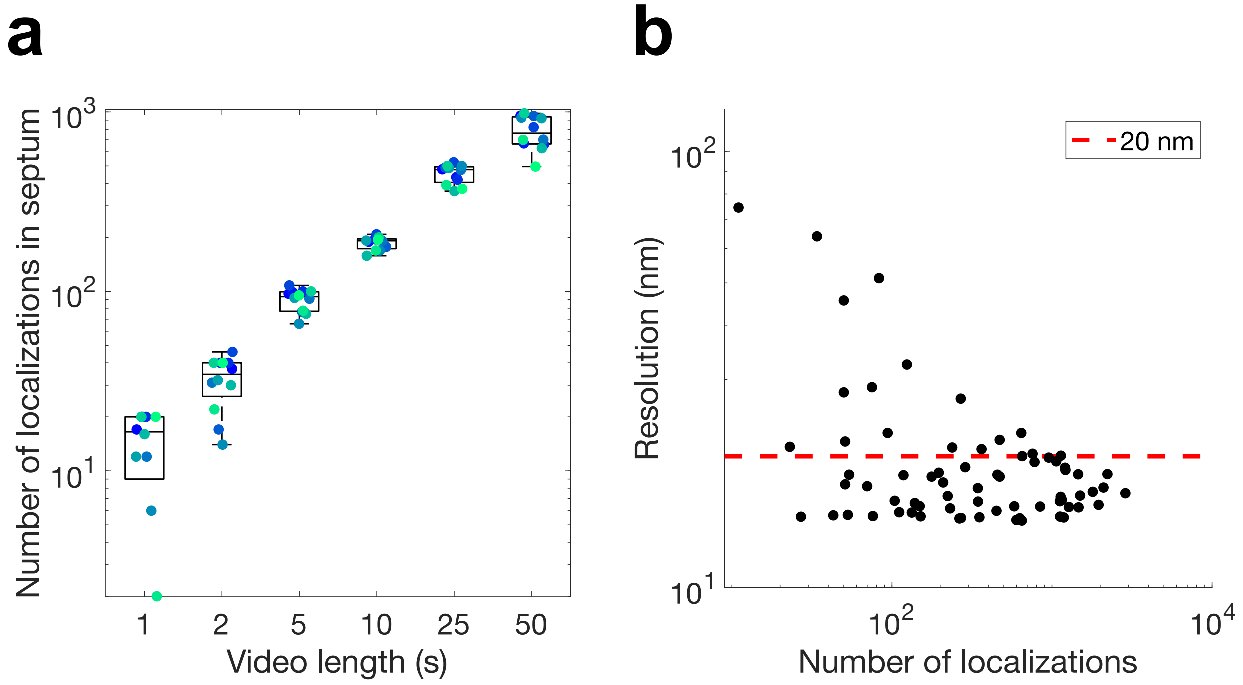

Supplementary Figure 2. Image resolution can reach 20 nm within five seconds while imaging Cdc12p-SYNZIP18 + SYNZIP17-mNG. 12 individual cells were imaged for 50 s (1,000 frames with 50 ms exposures). (a) Videos were truncated after the given number of seconds as shown. For each video the number of localizations in the septum is shown. Each cell is indicated by a different color dot, which can be tracked across the different video lengths. In the box, the middle line represents the median, the bottom line represents the 25th percentile, and the bottom line represents the 75th percentile. The whiskers reach to the furthest points in the data which are not outliers. (b) Resolution was calculated for each of the points in (A) and shown as a function of the number of localizations in the septum (as described in Methods). Cells were grown in media containing 0.005% galactose.

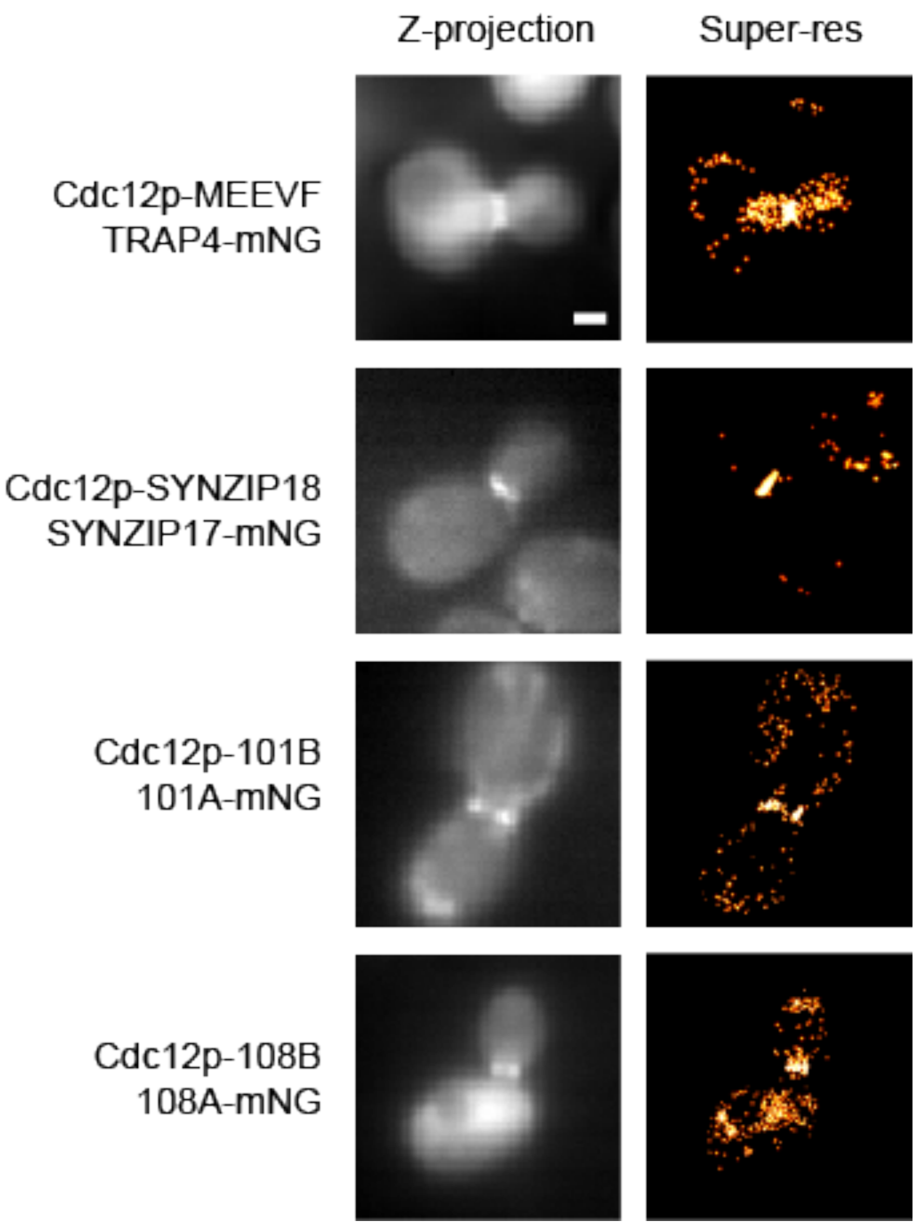

Supplementary Figure 3. LIVE-PAINT can be performed with different peptide-protein interaction pairs. The interaction pairs, TRAP4-MEEVF, SYNZIP17-SYNZIP18, 101A-101B, and 108A-108B, were used to tag Cdc12p and imaged, as indicated. (Left) Z-projections showing the average fluorescence signal for each video, calculated by integrating the average intensity over the entire video. (Right) Super-resolution images for each video. All images were constructed from videos collected for 1,000 frames, with

a 50 ms exposure per frame. Number of localization events: TRAP4-MEEVF: 429; SYNZIP17-SYNZIP18: 398; 101A-101B: 582; 108A-108B: 803. Images were recorded with an exposure time of 50 ms with a laser power density of 3.1 W/cm^2. Scale bars are 1 μm.

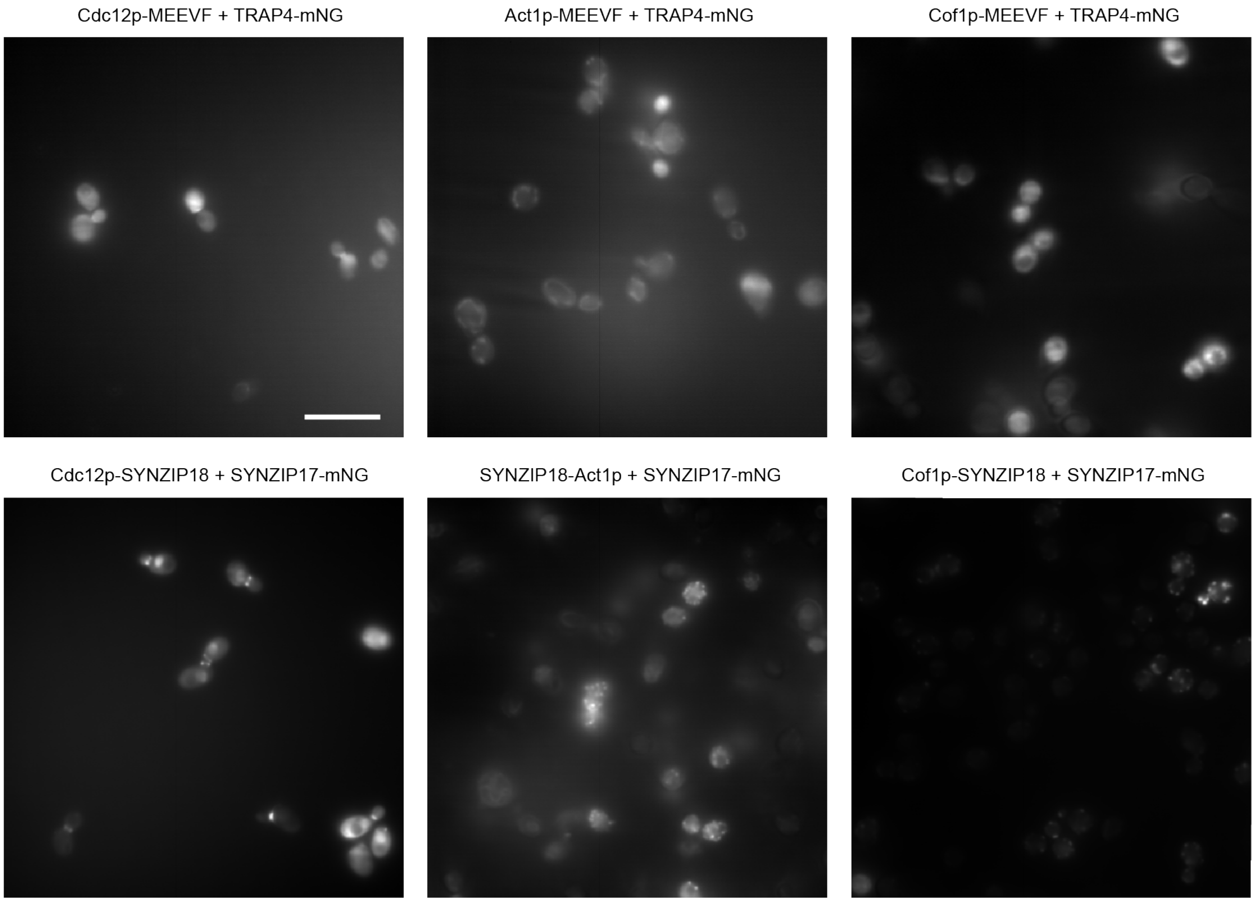

Supplementary Figure 4. TRAP4-MEEVF and SYNZIP17-SYNZIP18 interactions are specific inside the cell. Maximum projection images of Cdc12p, Act1p, and Cof1p, which were tagged by either the TRAP4-MEEVF (top row) or SYNZIP17-SYNZIP18 (bottom row) interaction pair. Structures localized to the septum are seen when tagging Cdc12 and puncta around the edge of the cell are observed when tagging actin of Cof1. All images were constructed from videos collected for 1,000 frames, with a 50 ms exposure per frame and a laser power density of 3.1 W/cm^2. Scale bar is 10 μm.

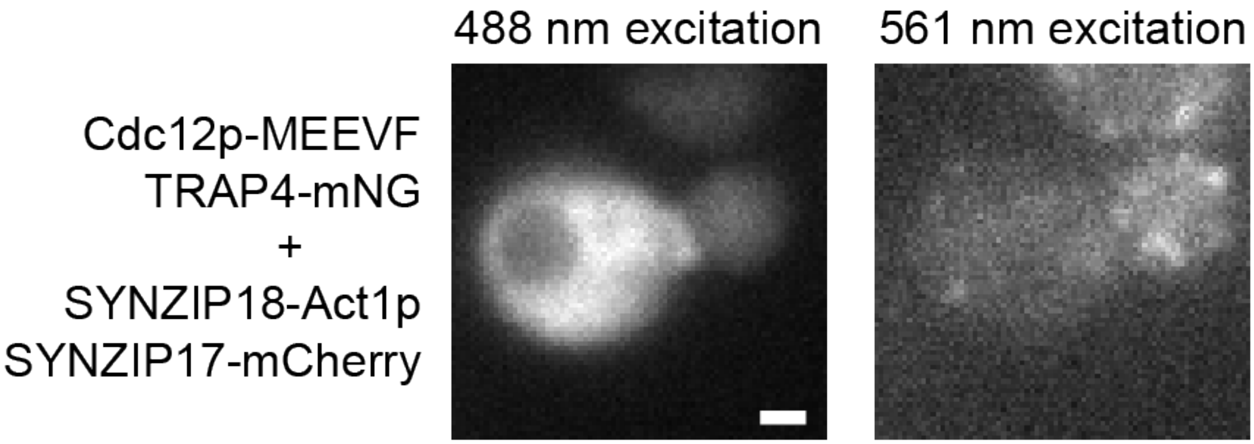

Supplementary Figure 5. TRAP4-MEEVF and SYNZIP17-SYNZIP18 interaction pairs are orthogonal to one another and can be used with two different fluorescent proteins for concurrent imaging. Fluorescence images of a cell expressing both Cdc12p-MEEVF+TRAP4-mNG and SYNZIP18-Act1p+SYNZIP17-mCherry. (Left) Cell imaged using a 488 nm excitation laser and green emission filter. Structure at yeast septum, corresponding to the location of Cdc12p, is clearly visible. mNG fluorescence would be detected using these excitation and emission settings. (Right) Cell imaged using a 561 nm excitation laser, using a power density of 50 W/cm^2. Distinctive structures around the edge of the cell, corresponding to the location of Act1p, are clearly visible. mCherry fluorescence would be detected using these excitation and emission settings. Images were collected using a 1 s exposure time. Scale bars are 1 μm.

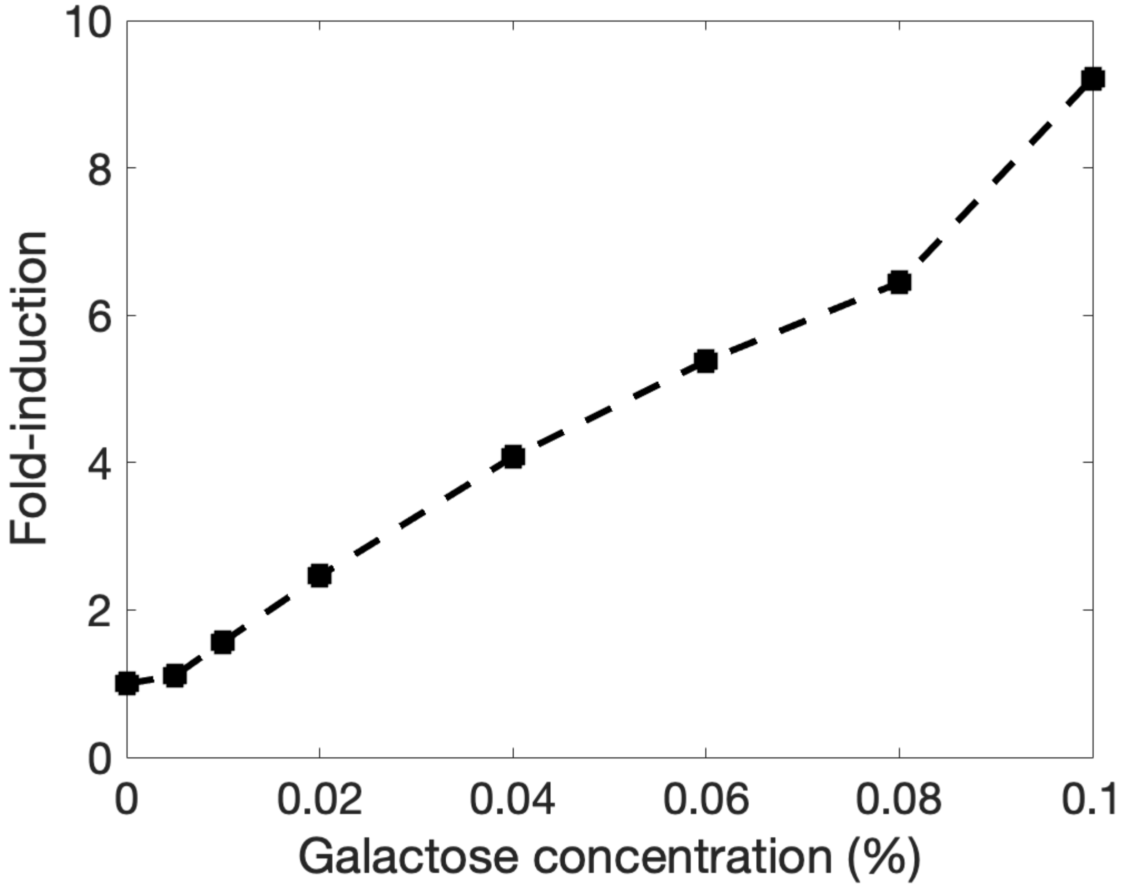

Supplementary Figure 6. Expression of mNG under the *GAL1* promoter is linear with galactose concentration in gal2Δ background. SYNZIP17-mNG was expressed under pGAL1 and grown overnight in synthetic complete media supplemented with 1% raffinose and a variable amount of galactose. No glucose was added to the media, as glucose represses the *GAL1* promoter. The expression of mNG was normalized first to the OD_{600} of the culture, which was between 0.12 and 0.16. This fluorescence value was then normalized to the expression level at 0% galactose. At higher galactose concentrations (2%) we have seen fold-induction values of ~30.

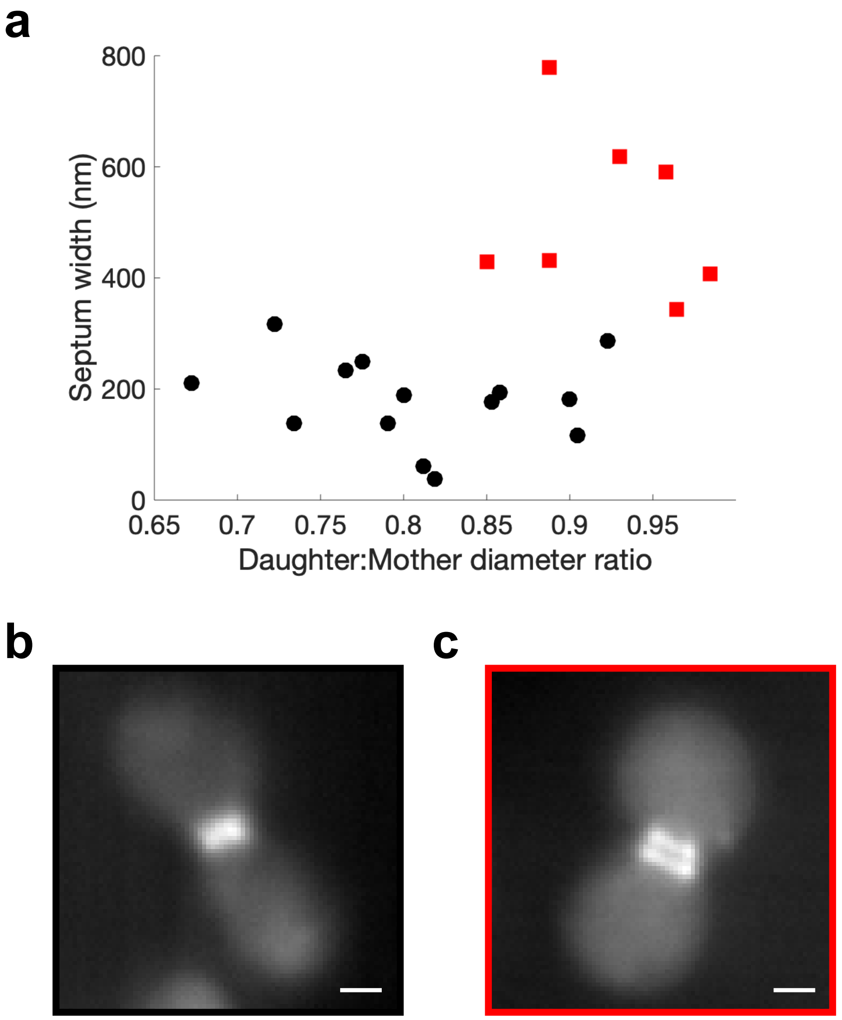

Supplementary Figure 7. Yeast septum width increases with daughter:mother diameter ratio. (a) septum width plotted as a function of yeast daughter:mother diameter ratio, with single-ring septa plotted with black dots and double-ring septa plotted with red squares. See methods for how we determined septum width. Single-ring and double-ring septa were readily identifiable from fluorescence images of single cells. (b) and (c) show representative fluorescence images for single-ring and double-ring septa, respectively. Both images were constructed from videos collected for 5,000 frames, with a 50 ms exposure per frame and a laser power density of 3.1 W/cm^2. Scale bars are 1 μm.

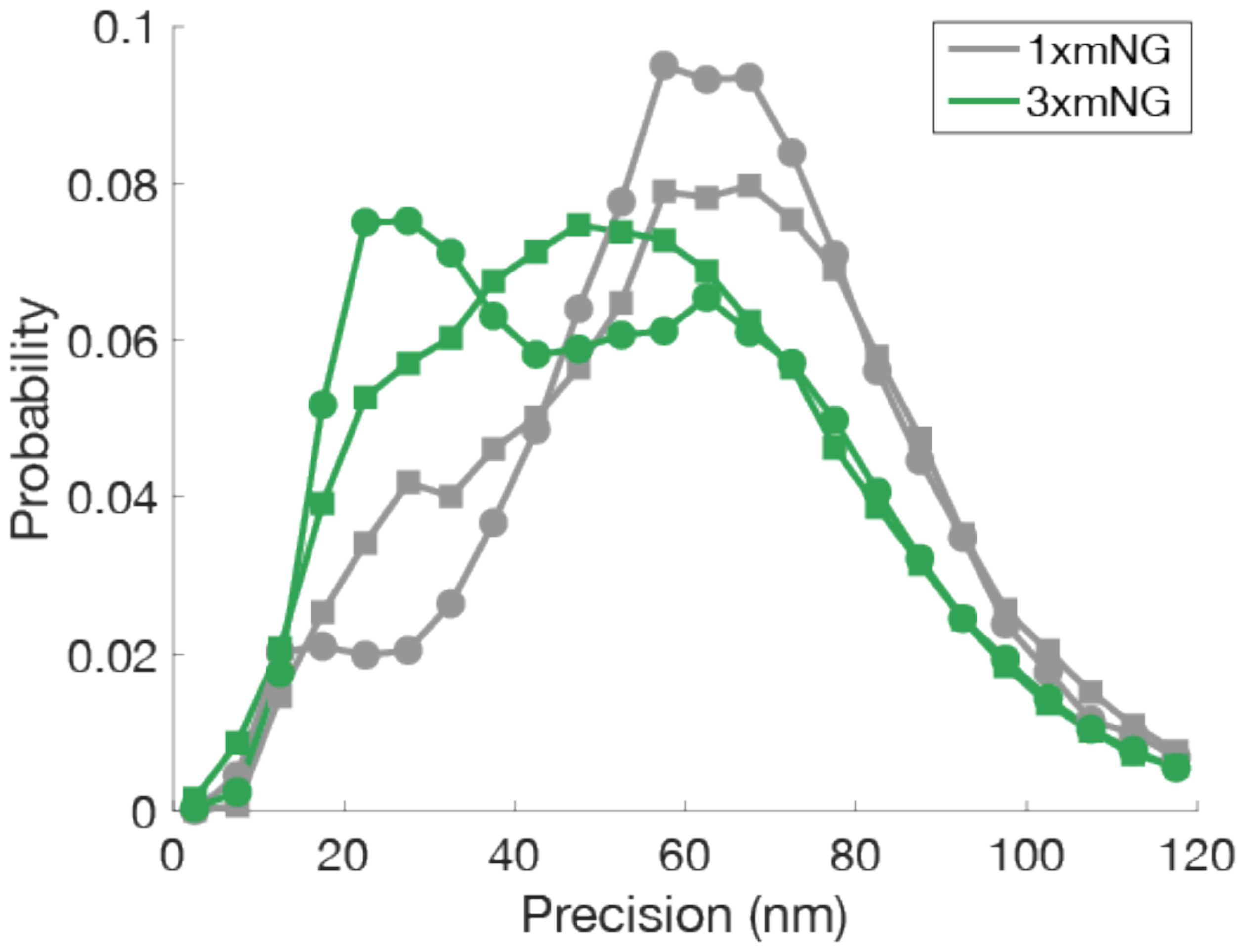

Supplementary Figure 8. SYNZIP17-3xmNG shows improved localization precision compared with SYNZIP17-1xmNG. Full distribution of localization precision shown for Cdc12p-SYNZIP18 + SYNZIP17-3xmNG (green line; circles and squares indicate two replicates) and Cdc12-SYNZIP18 + SYNZIP17-1xmNG (gray line; circles and squares indicate two replicates). Both experiments were performed by expressing the fluorescent protein construct using 0.005% galactose in the yeast growth media. The curves show the average of replicates for both the 3xmNG and 1xmNG constructs (n = 2 biological replicates), while the data points for both replicates are given as spots. Microscopy videos used to generate this data was collected for 4,000 frames, with a 50

ms exposure per frame and a laser power density of 3.1 W/cm^2. The mean precision values of the 1xmNG replicates (63.4 nm and 63.9 nm) is significantly different from the mean precision of the 3xmNG replicates (52.2 nm and 53.1 nm), a with p-value of 0.05 as determined by a one-tailed t-test (see methods). When comparing the medians between 1xmNG replicates (63.1 nm and 63.5 nm) and 3xmNG replicates (52.2 nm and 53.1 nm), we obtained a similar result, with a p-value of 0.038. The number of localizations events for each curve is: 1xmNG: 31503 (gray circles) and 28053 (gray squares); 3xmNG: 56269 (green circles) and 69565 (green squares).

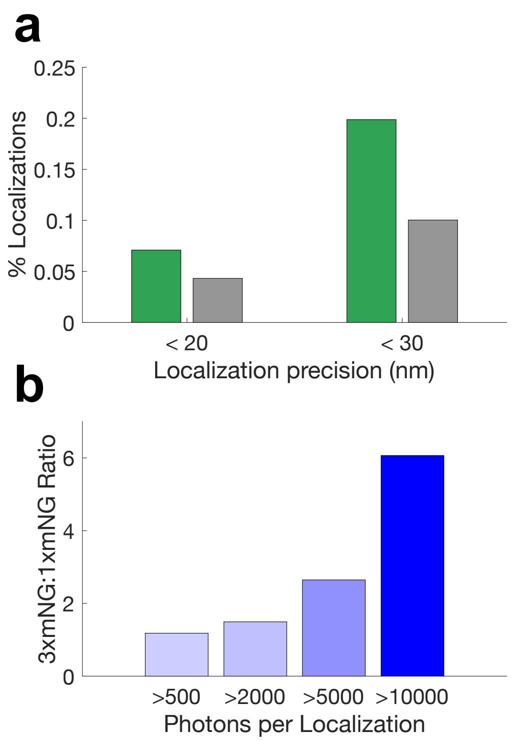

Supplementary Figure 9. Three tandem copies of mNG (3xmNG) shows improved localization precision compared with a single copy of mNG fused to the cognate peptide binding protein (a) Percentage of localizations with precision values < 20 nm or < 30 nm. Green bars represent data for 3xmNG, and the gray bars represents data for 1xmNG. (b) LIVE-PAINT with 3xmNG gives higher numbers of localizations with a large number of photons than with mNG. The 3xmNG:mNG ratio of number of localizations is plotted for each 'photons per localization' bin. The darker the blue bar, the greater the enrichment in probability for 3xmNG compared with mNG in that bin. Data was collected over a field of view including multiple cells for both the 1xmNG and 3xmNG data, and two technical replicates were collected. Microscopy videos used to generate this data was collected for 4,000 frames, with a 50 ms exposure per frame and a laser power density of 3.1 W/cm^2. This data was combined for this analysis. Number of localizations events: 1xmNG: 59556; 3xmNG: 125834.

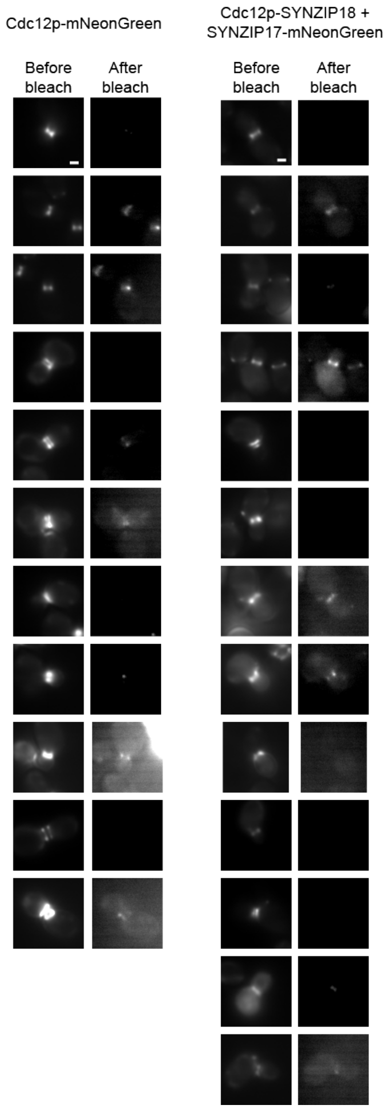

Supplementary Figure 10. Maximum projection images generated from videos collected before and after bleaching, for all cells analyzed for the data shown in Figure 3. (Left) Maximum projection images are shown for cells expressing Cdc12p-mNG. (Right) Maximum projection images are shown for cells expressing Cdc12p-SYNZIP18 + SYNZIP17-mNG. All images were constructed from videos collected for 1,000 frames, with a 50 ms exposure per frame and a laser power density of 3.1 W/cm^2. Scale bar is 1 µm.

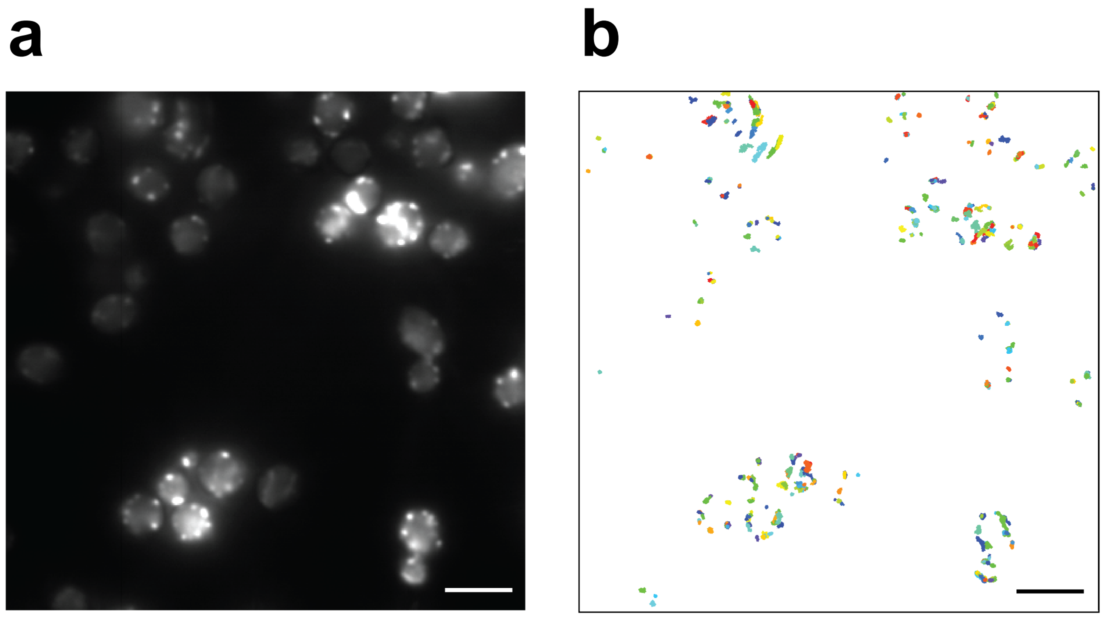

Supplementary Figure 11. Tracking of Cof1p in yeast cells. (a) Diffraction-limited image of the Cof1p in the yeast cells. (b) Tracks from individual Cof1p clusters. The image was constructed from a video collected for 4,000 frames, with a 50 ms exposure per frame and a laser power density of 3.1 W/cm^2. Scale bar is 5 μm.

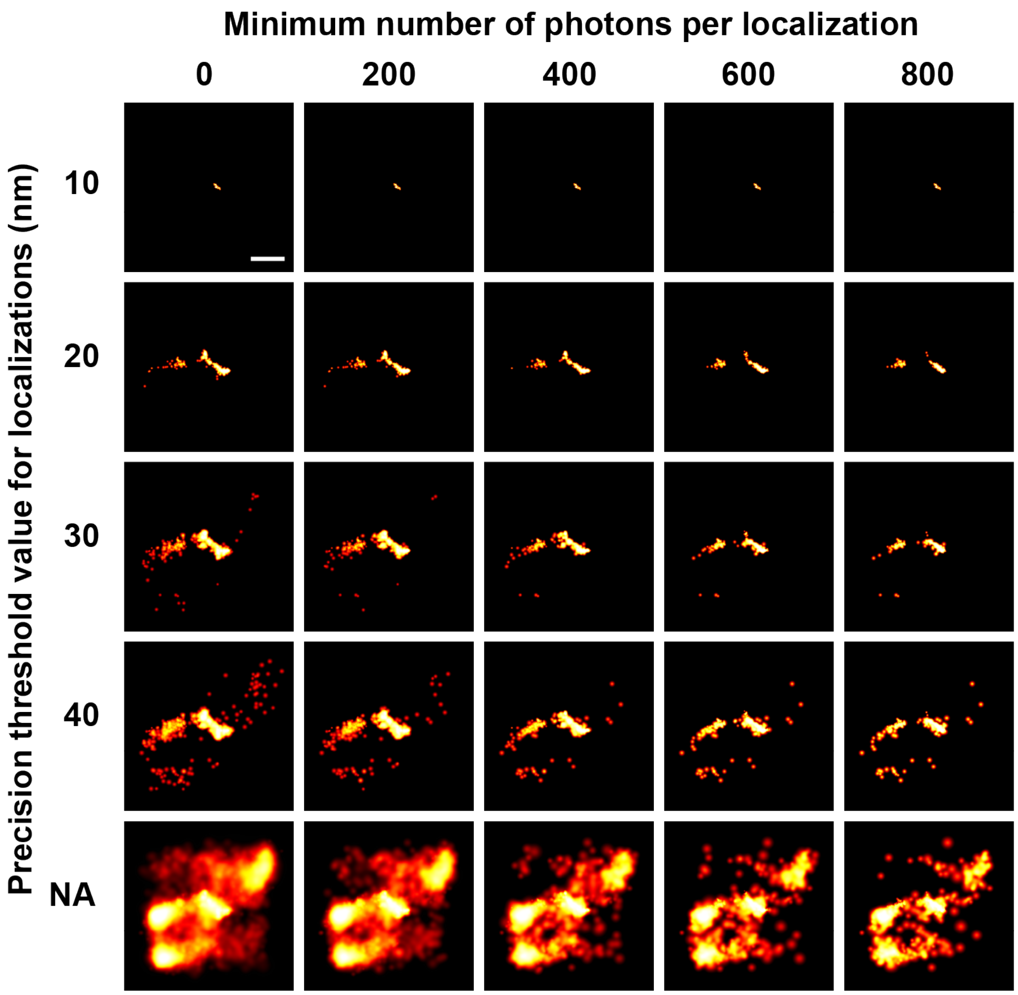

Supplementary Figure 12. One stack of images analyzed using different thresholds for localization precision and minimum number of photons per localization. NA indicates no precision value specified. The video used to construct the images collected for 6,000 frames, with a 50 ms exposure per frame and a laser power density of 3.1 W/cm^2. Number of localizations for each precision value, from left to right in the image: 10 nm: 46, 46, 46, 46, 46; 20 nm: 633, 617, 497, 328, 247; 30 nm: 1386, 1169, 746, 438, 330; 40 nm: 2013, 1547, 887, 526, 392; NA: 6248, 3764, 1855, 1089, 724. Scale bar is 1 μm.

Supplementary Tables

Supplementary Table 1. Quantification of the total number of super-resolution localisations and the percentage of these localisations in the septum for images shown in Figure 2.

	**TRAP**		**SYNZIP**	
Galactose concentration	% localisations in septum	Total number of localisations	% localisations in septum	Total number of localisations
0 %	14.71	272	94.12	153
0.005 %	44.54	348	97.99	398
0.02%	37.76	429	42.69	878
0.1%	22.78	1203	18.51	1313

Supplementary Table 2. Sequencing Primers

Label	Name	Sequence	Purpose
S1	CDC12_CT_F	GAGGGTCACGAGAACACC	Check C-terminus of *CDC12*
S2	CDC12_CT_R	CAGTTACTTCTGCTGGTTCC	Check C-terminus of *CDC12*
S3	GAL2_Seq_F	CTAATCCAAGGAGGTTTAC	Check *GAL2* locus
S4	GAL2_Seq_R	TAAGAGAGATGATGGAGC	Check *GAL2* locus
S5	SP6_Seq_F	ATTTAGGTGACACTATAG	Sequence pFA6a-HIS3MX6 and pFA6a-KANMX6 plasmids
S6	T7_Seq_F	TAATACGACTCACTATAGGG	Sequence pFA6a-HIS3MX6 and pFA6a-KANMX6 plasmids
S7	HIS_SEQ_F	CGTTAGAACGCGGCTAC	Sequence pFA6a-HIS3MX6 plasmids
S8	GAL2_SEQ_F2	GCTGCAGAAGGCACATCTA	Check *GAL2* locus
S9	GAL2_SEQ_R2	CCCAGAGATAAGTCTGGTGATG	Check *GAL2* locus
S10	pCUP1_seq_F	CATATAGAAGTCATCGACTAGT	Check pCu415CUP1 plasmid
S11	pCUP1_seq_R	GACGGTATCGATAAGCTT	Check pCu415CUP1 plasmid
S12	COF1_seq_F	CCTTAAACGGTGTCTCTACC	Check C-terminus of *COF1*

S13	COF1_seq_R	GGTGTACGGGACCTTAAATC	Check C-terminus of *COF1*

Supplementary Table 3. Primers for amplifying plasmid backbone

Label	Name	Sequence	Purpose
C1	p6k_ath1_F	TTGCAAACCAGAGCCTG	Amplify pFA6a-KANMX6 backbone to replace MEEVF with another peptide
C2	p6k_ath1_R	TGATGAGTCATGTAATTAGTTATGT	Amplify pFA6a-KANMX6 backbone to replace MEEVF with another peptide
C3	p6h_ath2_F	GAATCCGGGGTTTTTTCT	Amplify pFA6a-HIS3MX6 backbone to replace TRAP4 with another protein
C4	p6h_ath2_R	CTGCAGATGAGTGCGATTA	Amplify pFA6a-HIS3MX6 backbone to replace TRAP4 with another protein
C5	p6h_ath3_t4_F	TTGCTCCTTCAGGATTTTCT	Amplify pFA6a-HIS3MX6 backbone to replace TRAP4 mEOS with another fluorescent protein (e.g. mNG). TRAP4 is left to make a TRAP4-fluorescent protein fusion.
C6	p6h_ath3_sz_F	CTTGTAAGCTTCAATTTCCTTTCTCAAGT	Amplify pFA6a-HIS3MX6 backbone to replace

			SYNZIP17 mEOS with another fluorescent protein (e.g. mNG). SYNZIP17 is left to make a SYNZIP17-fluorescent protein fusion.
C7	p6h_ath3_R	TGATAAGTCATGTAATTAGTTATGTC	Amplify pFA6a-HIS3MX6 backbone to replace TRAP4 or SYNZIP mEOS with another fluorescent protein (e.g. mNG). TRAP4 or SYNZIP17 is left to make a TRAP4/SYNZIP17-fluorescent protein fusion.
C8	pCUP1_ATH_F	AAGCTTATCGATACCGTC	Amplify pCu415CUP1 backbone to replace product under expression of *CUP1* promoter
C9	pCUP1_ATH_R	ACTAGTCGATGACTTCTATATG	Amplify pCu415CUP1 backbone to replace product under expression of *CUP1* promoter

Supplementary Table 4. Integration primers

Label	Name	Sequence	Purpose
I1	p6h_int_F	CTAATCCAAGGAGGTTTACGGACCAGGGGAACTTTCCAGATTCAGAAGCTTCGTACGCTGCA	Amplify the entire cassette from pFA6a-HIS3MX6 (e.g. TRAP4-mEOS under *GAL1* promtoer, plus *HIS3* marker) for transformation into yeast
I2	p6h_int_R	CATGAAAAATTAAGAGAGATGATGGAGCGTCTCACTTCAAACGCAGGCGTTAGTATCGAATCG	Amplify the entire cassette from pFA6a-HIS3MX6 (e.g. TRAP4-mEOS under *GAL1* promoter, plus *HIS3* marker) for transformation into yeast
I3	p6k_int_F	GAAGAGCAGGTCAAAAGCTTGCAAGTAAAAAAATCCCATTTAAAAGGTGGATCAGGCTCTGG	Amplify the entire cassette from pFA6a-KANMX6 (e.g. GS-MEEVF, plus KANMX6 marker) for transformation into yeast
I4	p6k_int_R	AGGCGTTGAAATTGACGAGACAAAGAGGAAGA	Amplify the entire cassette from pFA6a-KANMX6 (e.g. GS-MEEVF, plus KANMX6 marker) for transformation into yeast

		CATTAATTAATCATTAGAAAAACTCATCGAGCATC	
I5	p6kcof1intF	TACGATTCTGTTTTGGAAAGAGTCAGCAGAGGCGCTGGTTCTCATGGTGGATCAGGCTCTGG	Amplify the entire cassette from pFA6a-KANMX6 (e.g. GS-MEEVF, plus KANMX6 marker) for transformation into yeast at *COF1* locus
I6	p6kcof1intR	TTTCATTTTTCTTGAAGATTGTTGTCATTTGTGAAATCATTTACCATTAGAAAAACTCATCGAGCATC	Amplify the entire cassette from pFA6a-KANMX6 (e.g. GS-MEEVF, plus KANMX6 marker) for transformation into yeast at *COF1* locus

Supplementary Table 5. Yeast strains used in this study

Strain	Parent	Genotype	Reference
BY4741	-	MATa his3Δ1 leu2Δ0 met15Δ0 ura3Δ0	Marsden Lab
TRAP4-mNG	BY4741	MATa his3Δ1 leu2Δ0 met15Δ0 ura3Δ0 gal2Δ::His3MX6 TRAP4-mNG	This study
SYNZIP17-mNG	BY4741	MATa his3Δ1 leu2Δ0 met15Δ0 ura3Δ0 gal2Δ::His3MX6 SYNZIP17-mNG	This study
CDC12-MEEVF + TRAP4-mNG	BY4741	MATa his3Δ1 leu2Δ0 met15Δ0 ura3Δ0 gal2Δ::His3MX6 TRAP4-mNG CDC12-MEEVF::KanMX6	This study
CDC12-MEEVF + TRAP4-mNG + SYNZIP17-mCherry	BY4741	MATa his3Δ1 leu2Δ0 met15Δ0 ura3Δ0: Ura3 SYNZIP17-mCherry gal2Δ::His3MX6 TRAP4-mNG CDC12-MEEVF::KanMX6	This study
CDC12-SYNZIP18 + SYNZIP17-mNG	BY4741	MATa his3Δ1 leu2Δ0 met15Δ0 ura3Δ0 gal2Δ::His3MX6 SYNZIP17-mNG CDC12-SYNZIP18::KanMX6	This study
CDC12-SYNZIP18 + SYNZIP17-mKO	BY4741	MATa his3Δ1 leu2Δ0 met15Δ0 ura3Δ0 gal2Δ::His3MX6 SYNZIP17-mKO CDC12-SYNZIP18::KanMX6	This study

CDC12-SYNZIP18 + SYNZIP17-mOrange	BY4741	MATa his3Δ1 leu2Δ0 met15Δ0 ura3Δ0 gal2Δ::His3MX6 SYNZIP17-mOrange CDC12-SYNZIP18::KanMX6	This study
CDC12-SYNZIP18 + SYNZIP17-mCherry	BY4741	MATa his3Δ1 leu2Δ0 met15Δ0 ura3Δ0 gal2Δ::His3MX6 SYNZIP17-mCherry CDC12-SYNZIP18::KanMX6	This study
CDC12-SYNZIP18 + SYNZIP17-3xmNG	BY4741	MATa his3Δ1 leu2Δ0 met15Δ0 ura3Δ0 gal2Δ::His3MX6 SYNZIP17-3xmNG CDC12-SYNZIP18::KanMX6	This study
CDC12-101B + 101A-mNG	BY4741	MATa his3Δ1 leu2Δ0 met15Δ0 ura3Δ0 gal2Δ::His3MX6 101A-mNG CDC12-101B::KanMX6	This study
CDC12-108B + 108A-mNG	BY4741	MATa his3Δ1 leu2Δ0 met15Δ0 ura3Δ0 gal2Δ::His3MX6 108A-mNG CDC12-108B::KanMX6	This study
CDC12-CCBN3,5 + CCAN3,5-mEOS	BY4741	MATa his3Δ1 leu2Δ0 met15Δ0 ura3Δ0 gal2Δ::His3MX6 CCAN3,5-mEOS CDC12-CCBN3,5::KanMX6	This study
COF1-MEEVF + TRAP4-mNG	BY4741	MATa his3Δ1 leu2Δ0 met15Δ0 ura3Δ0 gal2Δ::His3MX6 TRAP4-mNG COF1-MEEVF::KanMX6	This study

COF1-SYNZIP18 + SYNZIP17-mNG	BY4741	MATa his3Δ1 leu2Δ0 met15Δ0 ura3Δ0 gal2Δ::His3MX6 SYNZIP17-mNG COF1-SYNZIP18::KanMX6	This study

158
