# Supplementary material for: LIVE-PAINT allows super-resolution microscopy inside living cells using reversible peptide-protein interactions
Source: Commun Biol. 2020 Aug 20;3:458. doi: 10.1038/s42003-020-01188-6 (PMC7441314; doi:10.1038/s42003-020-01188-6)
Supplement: Supplementary file 6 — Reporting Summary [file 42003_2020_1188_MOESM6_ESM.pdf]

nature research

Corresponding author(s): Lynne Regan and Mathew Horrocks

Last updated by author(s): July 16, 2020

Reporting Summary

Nature Research wishes to improve the reproducibility of the work that we publish. This form provides structure for consistency and transparency in reporting. For further information on Nature Research policies, see Authors & Referees and the Editorial Policy Checklist.

Statistics

For all statistical analyses, confirm that the following items are present in the figure legend, table legend, main text, or Methods section.

n/a	Confirmed	
☐	☒	The exact sample size (*n*) for each experimental group/condition, given as a discrete number and unit of measurement
☐	☒	A statement on whether measurements were taken from distinct samples or whether the same sample was measured repeatedly
☐	☒	The statistical test(s) used AND whether they are one- or two-sided<br>*Only common tests should be described solely by name; describe more complex techniques in the Methods section.*
☒	☐	A description of all covariates tested
☒	☐	A description of any assumptions or corrections, such as tests of normality and adjustment for multiple comparisons
☒	☐	A full description of the statistical parameters including central tendency (e.g. means) or other basic estimates (e.g. regression coefficient) AND variation (e.g. standard deviation) or associated estimates of uncertainty (e.g. confidence intervals)
☒	☐	For null hypothesis testing, the test statistic (e.g. *F*, *t*, *r*) with confidence intervals, effect sizes, degrees of freedom and *P* value noted<br>*Give P values as exact values whenever suitable.*
☒	☐	For Bayesian analysis, information on the choice of priors and Markov chain Monte Carlo settings
☒	☐	For hierarchical and complex designs, identification of the appropriate level for tests and full reporting of outcomes
☒	☐	Estimates of effect sizes (e.g. Cohen's *d*, Pearson's *r*), indicating how they were calculated

Our web collection on statistics for biologists contains articles on many of the points above.

Software and code

Policy information about availability of computer code

Data collection	ImageJ was used to collect images and videos on the microscope.
Data analysis	Data analysis was performed in the Java 8 2017 release of Fiji. The GDSC SMLM plugin was used to produce both diffraction limited and super-resolution images and calculate quantitative imaging values, including resolution precision, signal to background, etc. MATLAB (release 2019b) was used to generate the plots used in the study. Functions in MATLAB (release 2019b) and Python (version 2.7.16) were used to perform Fourier Ring Correlation, diffusion analysis, and Feret distance analysis. A one-tailed t-test was performed using the ttest function in MATLAB.

For manuscripts utilizing custom algorithms or software that are central to the research but not yet described in published literature, software must be made available to editors/reviewers. We strongly encourage code deposition in a community repository (e.g. GitHub). See the Nature Research guidelines for submitting code & software for further information.

Data

Policy information about availability of data

All manuscripts must include a data availability statement. This statement should provide the following information, where applicable:

- Accession codes, unique identifiers, or web links for publicly available datasets
- A list of figures that have associated raw data
- A description of any restrictions on data availability

Data files used to generate all charts and graphs, as well as uncompressed supplementary videos, have been deposited to Edinburgh DataShare (DOIs: https://doi.org/10.7488/ds/2859 and https://doi.org/10.7488/ds/2801). A step-by-step protocol is available at Protocol Exchange (DOI: http://dx.doi.org/10.21203/rs.3.pex-1043/v1). All relevant data are available from the corresponding author upon reasonable request.

Field-specific reporting

Please select the one below that is the best fit for your research. If you are not sure, read the appropriate sections before making your selection.

☒ Life sciences ☐ Behavioural & social sciences ☐ Ecological, evolutionary & environmental sciences

For a reference copy of the document with all sections, see nature.com/documents/nr-reporting-summary-flat.pdf

Life sciences study design

All studies must disclose on these points even when the disclosure is negative.

Sample size	No sample size calculation was performed. The sample sizes used in this study are sufficient, because we make only one statistical claim about the difference between two populations and calculate an appropriate p-value to demonstrate significance. In this work we are presenting the feasiblility of a new method.
Data exclusions	Data were excluded from analysis of the septum when the cells being studied did not show any septum structure by eye. This is because we analyze the separation of septal rings during cell division, so only dividing cells showing a septum structure were of interest.
Replication	Samples were prepared and imaged on different days, including realignment of the microscope. All our findings were reproducible.
Randomization	This is not relevant to our study. We used different yeast strains with known genomic integrations, so our sample groups were self-evident.
Blinding	Blinding was not relevant to our study, because we did not use human research participants or animal models, only yeast.

Reporting for specific materials, systems and methods

We require information from authors about some types of materials, experimental systems and methods used in many studies. Here, indicate whether each material, system or method listed is relevant to your study. If you are not sure if a list item applies to your research, read the appropriate section before selecting a response.

Materials & experimental systems

n/a	Involved in the study	
☒	☐	Antibodies
☒	☐	Eukaryotic cell lines
☒	☐	Palaeontology
☒	☐	Animals and other organisms
☒	☐	Human research participants
☒	☐	Clinical data

Methods

n/a	Involved in the study	
☒	☐	ChIP-seq
☒	☐	Flow cytometry
☒	☐	MRI-based neuroimaging
