# Supplementary material for: LIVE-PAINT allows super-resolution microscopy inside living cells using reversible peptide-protein interactions
Source: Commun Biol. 2020 Aug 20;3:458. doi: 10.1038/s42003-020-01188-6 (PMC7441314; doi:10.1038/s42003-020-01188-6)
Supplement: Supplementary file 8 — Description of Additional Supplementary Files [file 42003_2020_1188_MOESM8_ESM.pdf]

Description of Additional Supplementary Files

File Name: **Supplementary Movie 1**
Description: Video showing blinking of mKO during LIVE-PAINT data acquisition.

File Name: **Supplementary Movie 2**
Description: Video showing blinking of mOrange during LIVE-PAINT data acquisition.

File Name: **Supplementary Movie 3**
Description: Video showing moving cofilin clusters.

File Name: **Supplementary Movie 4**
Description: Video showing tracks for moving cofilin clusters.
